# Supplementary material for: Effect of Prophylactic Amiodarone Infusion on the Recurrence of Ventricular Arrhythmias in Out-of-Hospital Cardiac Arrest Survivors: A Propensity-Matched Analysis
Source: J Clin Med. 2019 Feb 13;8(2):244. doi: 10.3390/jcm8020244 (PMC6406616; doi:10.3390/jcm8020244)
Supplement: Supplementary file 1 [file jcm-08-00244-s001.pdf]

Table S1. Cardiopulmonary resuscitation profiles and post-cardiac arrest care between the non-prophylactic amiodarone and prophylactic amiodarone groups.

Variables	No Prophylactic Amiodarone (*n* = 320)	Prophylactic Amiodarone (*n* = 124)	*p* Value
Witnessed	265 (82.8)	99 (79.8)	0.46
Bystander CPR	201 (62.8)	63 (50.8)	0.07
Etiology of cardiac arrest			0.90
Presumed cardiac cause	279 (87.2)	110 (88.7)	
Respiratory cause	8 (2.5)	3 (2.4)	
Other medical condition	33 (10.3)	11 (8.9)	
Prehospital shockable rhythm	215 (67.2)	80 (64.5)	0.59
Prehospital defibrillation number	1.0 [0.0–2.0]	1.0 [0.0–2.0]	0.59
ED defibrillation number	1.0 [0.0–2.0]	1.5 [0.0–4.0]	0.006
CPR drugs			
Epinephrine	195 (63.7)	88 (73.3)	0.06
Vasopressin	11 (3.6)	7 (5.8)	0.31
Lidocaine	7 (2.3)	10 (8.3)	0.01
Magnesium	12 (3.9)	18 (15.0)	< 0.001
Bicarbonate	34 (11.1)	31 (25.8)	< 0.001
Amiodarone	62 (20.2)	53 (43.8)	< 0.001
ECMO CPR	11 (3.9)	4 (3.3)	0.50
No flow time, min	0.0 [0.0–6.0]	1.0 [0.0–6.0]	0.29
Low flow time, min	29.0 [20.0–40.0]	34.0 [22.0–48.0]	0.02
Electrocardiography			
ST-segment elevation	88 (27.5)	38 (30.6)	0.51
ST-segment depression	123 (38.4)	38 (30.6)	0.13
Left bundle branch block	30 (9.4)	7 (5.6)	0.20
Right bundle branch block	35 (10.9)	16 (12.9)	0.56
Normal ST and T wave	72 (22.5)	25 (20.2)	0.59
Prolonged QTc interval	191 (59.7)	89 (71.8)	0.02
Non-sustained VT	0 (0.0)	3 (2.4)	0.02
Ventricular premature complex	35 (10.9)	24 (19.4)	0.02
Coronary artery angiography	210 (65.6)	90 (72.6)	0.16
Left anterior descending stenosis	78 (37.1)	59 (65.6)	< 0.001
Right coronary artery stenosis	67 (31.9)	47 (52.2)	0.001
Left circumflex artery stenosis	61 (29.0)	45 (50.0)	0.001
Significant coronary stenosis			<0.001
1 vessel	56 (26.7)	20 (22.2)	
2 vessels	18 (8.6)	4 (4.4)	
3 vessels	38 (18.1)	41 (45.6)	
Number of coronary vessels	1.0 [0.0–2.0]	1.5 [0.0–3.0]	<0.001
Percutaneous coronary intervention	77 (24.1)	35 (28.2)	0.37
Left ventricular ejection fraction (%) during TTM, *n* = 217	*n* = 157, 55.0 [40.0–62.0]	*n* = 60, 52.2 [36.3–59.9]	0.39
Cardiovascular drugs during TTM			
Dopamine	186 (58.5)	98 (79.0)	< 0.001
Norepinephrine	172 (54.1)	80 (64.5)	0.05
Vasopressin	28 (8.8)	7 (5.6)	0.27
Epinephrine	28 (8.8)	7 (5.7)	0.28
Dobutamine	39 (12.3)	22 (17.9)	0.13

Values are expressed as medians [interquartile range] or numbers (%). Abbreviations: CPR, cardiopulmonary resuscitation; ED, emergency department; ECMO, extracorporeal membrane oxygenation; QTc, corrected QT segment; ROSC, return of spontaneous circulation; CAG, coronary artery angiography; TTM, target temperature management.

Table S2. Detailed outcome results.

Variables	No Prophylactic Amiodarone (n = 320)	Prophylactic Amiodarone (n = 124)	p Value
Recurrent shockable arrest	n = 29 (9.3)	n = 21 (16.9)	
Outcome of recurrent shockable arrhythmia			
ROSC	27 (93.1)	21 (100)	0.50
No ROSC	2 (6.9)	0	
Survival discharge	18 (62.1)	17 (81.0)	0.15
In-hospital death	11 (37.9)	4 (19.0)	
Causes of in-hospital death	n = 84 (26.3)	n = 26 (21.0)	0.17
Cardiovascular	28 (33.3)	13 (50.0)	
Cerebral	8 (9.5)	1 (3.8)	
Sepsis	28 (33.3)	10 (38.5)	
Others or undetermined	20 (23.8)	2 (7.7)	

Values are expressed as number (%). Abbreviations: ROSC, return of spontaneous circulation.

Table S3. Logistic regression analysis for recurrent shockable arrest rhythm.

Variables	Univariate Analysis				Multivariable Analysis			
	Odds Ratio	95% CI		p-Value	Odds Ratio	95% CI		p-Value
		Lower	Upper			Lower	Upper	
Age, year	1.006	0.987	1.025	0.55				
Male	0.942	0.481	1.845	0.86				
Laboratory findings, initial								
CK-MB, ng/mL	1.003	1.000	1.005	0.02				
CPR–related variables								
Witnessed	0.864	0.413	1.810	0.70				
Bystander CPR	1.175	0.636	2.173	0.61				
Presumed cardiac cause	1.042	0.422	2.572	0.93				
Prehospital initial rhythm								
Non-shockable	Reference							
Shockable	1.921	0.656	5.625	0.23				
Unknown	2.792	0.865	9.012	0.09				
Prehospital defibrillation number	0.883	0.687	1.134	0.33				
ED defibrillation number	0.987	0.901	1.081	0.77				
ED defibrillation energy, J	1.000	0.999	1.000	0.49				
Electrocardiography								
ST-segment elevation	1.811	0.986	3.327	0.06				
ST-segment depression	1.732	0.958	3.131	0.07				
Left bundle branch block	0.676	0.200	2.287	0.53				
Right bundle branch block	1.555	0.685	3.529	0.29				
Prolonged QTc interval	1.278	0.682	2.396	0.44				
Non–sustained VT	1.106	0.036	34.351	0.95				
Ventricular premature complex	3.409	1.725	6.738	<0.001	3.724	1.823	7.606	<0.001
Coronary artery angiography								
Interval of ROSC to CAG, h	1.000	0.999	1.001	0.73				
Left anterior descending stenosis	0.806	0.403	1.612	0.54				
Right coronary artery stenosis	0.574	0.263	1.255	0.16				
Left circumflex artery stenosis	0.535	0.237	1.206	0.13				
Number of significant coronary artery stenosis	0.820	0.598	1.123	0.22				
Percutaneous coronary intervention	1.047	0.535	2.049	0.89				
Prophylactic amiodarone	2.046	1.117	3.746	0.02	1.946	1.038	3.647	0.04
Left ventricular ejection fraction (%) during TTM, $n = 217$	1.008	0.982	1.034	0.56				
Cardiovascular drugs during TTM								
Dopamine	2.127	1.057	4.282	0.03				
Norepinephrine	2.095	1.095	4.008	0.03				
Vasopressin	1.013	0.342	2.998	0.98				
Epinephrine	2.568	1.096	6.015	0.03	3.742	1.535	9.120	0.004
Dobutamine	1.431	0.657	3.116	0.37				

All variables including demographics, prehospital and in–hospital CPR variables, medications, and initial laboratory and electrocardiographic findings were analyzed using a logistic regression model. Variables showing $p < 0.1$ in univariate analysis were entered into multiple logistic regression analysis. Abbreviations: CI, confidence interval; CK-MB, creatinine kinase MB fraction; CPR, cardiopulmonary resuscitation; ED, emergency department; QTc, corrected QT segment; VT, ventricular tachycardia; ROSC, return of spontaneous circulation; CAG, coronary artery angiography; TTM, target temperature management.

Table S4. Characteristics, tests, managements, and outcomes in propensity-score-matched groups.

Variables	Total (*n* = 186)	Prophylactic Amiodarone (*n* = 93)	No prophylactic Amiodarone (*n* = 93)	*p*-Value	Standardized Difference of Means
Age, year	53.4 ± 15.5	53.56 ± 15.57	53.27 ± 15.58	0.89	0.019
Male	138 (74.2)	67 (72.0)	71 (76.3)	0.49	0.098
Past medical history					
History of cardiac arrest	2 (1.1)	1 (1.1)	1 (1.1)	> 0.99	0.000
Acute coronary syndrome	37 (19.9)	17 (18.3)	20 (21.5)	0.58	0.081
Arrhythmia	16 (8.6)	9 (9.7)	7 (7.5)	0.62	0.077
Hypertension	54 (29.0)	27 (29.0)	27 (29.0)	> 0.99	0.000
Diabetes	34 (18.3)	17 (18.3)	17 (18.3)	> 0.99	0.000
Chronic pulmonary disease	6 (3.2)	2 (2.2)	4 (4.3)	0.41	0.122
Chronic renal disease	3 (1.6)	1 (1.1)	2 (2.2)	0.56	0.085
Liver cirrhosis	0 (0)	0 (0)	0 (0)	–	0.000
Malignancy	0 (0)	0 (0)	0 (0)	–	0.000
Vital signs					
Systolic pressure, mmHg	118.1 ± 31.4	118.5 ± 28.8	117.8 ± 33.9	0.89	0.020
Diastolic pressure, mmHg	74.2 ± 21.2	74.2 ± 20.9	74.2 ± 21.7	> 0.99	0.001
Pulse rate, beats/min	101.2 ± 27.3	100.7 ± 28.7	101.8 ± 26.1	0.76	0.039
Body temperature, °C	36.2 [35.3–36.7]	36.1 [35.2–36.6]	36.3 [35.5–36.8]	0.28	0.120
Laboratory findings, initial					
White blood cell, ◎10^3/μL	13.5 [10.8–17.8]	13.0 [10.3–18.1]	13.7 [11.5–17.6]	0.53	0.022
Hemoglobin, g/dL	14.2 ± 2.3	14.2 ± 2.3	14.2 ± 2.3	> 0.99	0.001
Sodium, mmol/L	140.0 [137.0–143.0]	140.0 [137.0–143.0]	140.0 [138.0–143.0]	> 0.99	0.032
Potassium, mmol/L	3.8 [3.4–4.4]	3.7 [3.3–4.4]	4.0 [3.5–4.4]	0.24	0.127
Calcium, mg/dL	8.05 [7.5–8.9]	8.2 [7.6–8.8]	7.9 [7.3–9.0]	0.12	0.025
Magnesium, mg/dL	2.3 [2.1–2.6]	2.3 [2.1–2.5]	2.2 [2.0–2.7]	0.92	0.011
Troponin–I, ng/mL	0.585 [0.120–4.900]	0.529 [0.100–3.746]	0.648 [0.163–5.280]	0.10	0.091
CK–MB, ng/mL	9.12 [3.29–37.10]	9.13 [2.25–39.40]	9.11 [3.90–33.75]	0.41	0.048
Witnessed	151 (81.2)	73 (78.5)	78 (83.9)	0.35	0.138
Bystander CPR	103 (55.4)	50 (53.8)	53 (57.0)	0.64	0.065
Arrest cause: presumed cardiac cause	167 (89.8)	82 (88.2)	85 (91.4)	0.49	0.107
Total defibrillation number	3 [1–4]	3 [1–4]	2 [1–5]	0.91	0.072
CPR drugs					
Epinephrine	126 (67.7)	64 (68.8)	62 (66.7)	0.75	0.046
Vasopressin	6 (3.2)	2 (2.2)	4 (4.3)	0.41	0.122
Lidocaine	6 (3.2)	4 (4.3)	2 (2.2)	0.41	0.122
Magnesium	17 (9.1)	9 (9.7)	8 (8.6)	0.78	0.037
Bicarbonate	27 (14.5)	13 (14.0)	14 (15.1)	0.83	0.031
Amiodarone	63 (33.9)	28 (30.1)	35 (37.6)	0.22	0.160
Low flow time, min	30.5 [22.0–46.0]	33.0 [20.0–45.0]	30.0 [23.0–46.0]	0.92	0.061
Electrocardiography					
ST–segment elevation	53 (28.5)	27 (29.0)	26 (28.0)	0.87	0.024
ST–segment depression	52 (28.0)	27 (29.0)	25 (26.9)	0.75	0.048
Left bundle branch block	12 (6.5)	7 (7.5)	5 (5.4)	0.56	0.088
Right bundle branch block	21 (11.3)	11 (11.8)	10 (10.8)	0.82	0.034
Normal ST and T wave	37 (19.9)	17 (18.3)	20 (21.5)	0.56	0.081
Prolonged QTc interval	126 (67.7)	66 (71.0)	60 (64.5)	0.34	0.138
Non–sustained VT	1 (0.5)	1 (1.1)	0 (0)	0.32	0.147
Ventricular premature complex	31 (16.7)	16 (17.2)	15 (16.1)	0.85	0.029
Cardiovascular drugs during TTM					
Dopamine	133 (71.5)	68 (73.1)	65 (69.9)	0.56	0.072
Norepinephrine	113 (60.8)	54 (58.1)	59 (63.4)	0.45	0.110
Vasopressin	9 (4.8)	3 (3.2)	6 (6.5)	0.26	0.151
Epinephrine	12 (6.5)	5 (5.4)	7 (7.5)	0.53	0.088
Dobutamine	25 (13.4)	13 (14.0)	12 (12.9)	0.84	0.032
Outcome					
Recurrent shockable arrest	25 (13.4)	14 (15.1)	11 (11.8)	0.51	
Survival discharge	41 (22.0)	20 (21.5)	21 (22.6)	0.87	
Good neurologic outcome	83 (44.6)	38 (40.9)	45 (48.4)	0.26	

Values are means ± standard deviation, medians [interquartile range], or numbers (%). Abbreviations: CK-MB, creatinine kinase MB fraction; CPR, cardiopulmonary resuscitation; QTc, corrected QT segment; VT, ventricular tachycardia; TTM, target temperature management.
